# Mercury Contamination in Fish and Its Effects on the Health of Pregnant Women and Their Fetuses, and Guidance for Fish Consumption—A Narrative Review

**DOI:** 10.3390/ijerph192315929

**Published:** 2022-11-29

**Authors:** Bojian Chen, Shiyuan Dong

**Affiliations:** 1 Food Science and Engineering, Haide College, Ocean University of China, Qingdao 266100, China; 2 College of Food Science and Technology, Ocean University of China, Qingdao 266003, China

**Keywords:** fish as food, mercury contamination, pregnant women, fetuses, health impacts

## Abstract

As a principal source of long-chain omega-3 fatty acids (3FAs), which provide vital health benefits, fish consumption also comes with the additional benefit of being rich in diverse nutrients (e.g., vitamins and selenium, high in proteins and low in saturated fats, etc.). The consumption of fish and other seafood products has been significantly promoted universally, given that fish is an important part of a healthy diet. However, many documents indicate that fish may also be a potential source of exposure to chemical pollutants, especially mercury (Hg) (one of the top ten chemicals or groups of chemicals of concern worldwide), and this is a grave concern for many consumers, especially pregnant women, as this could affect their fetuses. In this review, the definition of Hg and its forms and mode of entrance into fish are introduced in detail and, moreover, the bio-accumulation of Hg in fish and its toxicity and action mechanisms on fish and humans, especially considering the health of pregnant women and their fetuses after the daily intake of fish, are also reviewed. Finally, some feasible and constructive suggestions and guidelines are recommended for the specific group of pregnant women for the consumption of balanced and appropriate fish diets in a rational manner.

## 1. Introduction

In recent years, the consumption of fish and other seafood has been significantly promoted universally, given that fish is an important part of a healthy diet in modern society, and is a major source of healthy long-chain omega-3 fatty acids (3FAs), mainly eicosapentaenoic acid (EPA) and docosahexaenoic acid (DHA). It is also rich in many nutrients, such as vitamins and selenium, high in proteins, and low in saturated fats, etc. [1,2,3,4,5]. Additionally, fish and other seafood are recommended to pregnant women, children, and the aged. However, many documents indicate that fish may also be a potential source of exposure to chemical pollutants (especially mercury (Hg) contamination) [6,7,8], and this is a concern for many consumers, especially pregnant women, as this could affect their fetuses [9,10,11,12,13]. Therefore, fish have health benefits and also contain contaminants, resulting in confusion over the role of fish consumption in a healthy diet.

Fish and shellfish are marine food products that contain detectable traces of elements, including Hg [14]. Hg (also known as hydrargyrum) can bio-accumulate in human bodies, primarily by consuming seafood, freshwater fish, shellfish, etc. [15], and can cause damage or harm to the healthy status of pregnant women and their fetuses. For the specific group of pregnant women and their fetuses, questions remain as to how about Hg contamination in fish and other seafood can be seen as part of a healthy diet. Additionally, what are the effects of Hg contamination in these marine foods on the health of pregnant women and their fetuses? In this review, the objectives were to introduce the forms and ways that Hg enters fish, as well as outline the bio-accumulation of Hg in fish, and its toxic impacts and action mechanisms on the health of pregnant women and their fetuses after consuming fish daily. Finally, some feasible and constructive suggestions are given to guide the specific group of pregnant women to select fish diets in a healthy manner.

## 2. Definition of Mercury (Hg) and Its Forms in Nature and Fish Bodies

**Figure 1:**
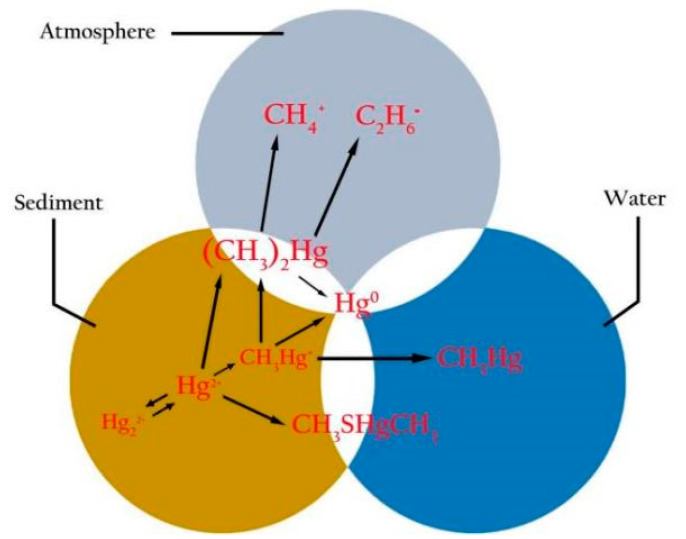
Existing forms and transformation of elemental, inorganic and organic mercury (Hg) in sediment, water and atmosphere.

Mercury (Hg), a cumulative neurotoxin that is present in the environment through a variety of natural and anthropogenic sources, is of grave concern because of its cellular, cardiovascular, hematological, pulmonary, renal, immunological, neurological, endocrine, reproductive, and embryonic toxicological impacts, etc. [16,17,18,19,20,21,22]. Hg is a well-established and cumulatively neurotoxic agent that can have serious adverse effects on the development and functioning of the human central nervous system (CNS), and it is environmentally ubiquitous [20]. In nature, Hg is unevenly distributed in the marine environment, and exists as three chemical forms, i.e., elemental Hg (metallic Hg^0^), inorganic Hg (Hg^+^ and Hg^2+^ as salts) and organic Hg (MeHg: CH_3_Hg^+^, etc.), which exist in sediment, water and the atmosphere (seen in Figure 1) [20,21,22,23,24,25]. In general, vapor Hg^0^ is readily absorbed from the lungs, and it can pass the blood–brain barrier (BBB) and placenta, resulting in high neurotoxicity, while liquid Hg^0^ is slightly absorbed from the gastrointestinal (GI) tract and does not appear to be toxic; inorganic Hg is concentrated in the kidneys and it cannot pass the BBB and placenta; and organic Hg is easily absorbed from the GI tract and it can pass the BBB, resulting in higher toxicity [26].

Hg circulates in aquatic systems in several ways [25]. On the one hand, anthropogenic activities (such as the use of Hg pesticides in agriculture and industrial waste) transfer Hg into groundwater through the soil layer, and Hg-containing wastes enter surface waters such as reservoirs through surface runoff, entering rivers, lakes or marine aquatic ecosystems. On the other hand, some natural causes can also carry elemental Hg into aquatic systems, and Hg that is naturally accumulated in atmospheric air and industrial emissions also enters into the atmosphere, eventually directly accumulating in surface waters, oceans, and other aquatic systems through wet and dry deposition. Moreover, the most common flow route of Hg deposition into marine ecosystems is standing surface waters, such as rivers, streams, and estuaries [27]. Hg is released from a variety of natural and anthropogenic sources into the three phases of natural water bodies, i.e., solid, aqueous and biological phases; it exists mainly in the forms of Hg^2+^, Hg(OH)_2_, CH_3_Hg^+^, CH_3_Hg(OH), CH_3_HgCl, and C_6_H_5_Hg^+^ in the aqueous phase, in the forms of Hg^+^, Hg^0^, HgO, HgS, CH_3_Hg(SR), and (CH_3_Hg)_2_S in the solid phase, and in the forms of Hg^2+^, CH_3_Hg^+^, and CH_3_HgCH_3_ in the biological phase. In natural water bodies, highly toxic methylmercury (MeHg) transformed through some hydrophytic microorganisms (i.e., bacteria) is a major source of Hg exposure to the general population of fish, and causes bio-magnification which disrupts the aquatic food web [28]. Moreover, it is presumed that MeHg is the predominant form of Hg transmission through food chains [29,30,31]. Harris et al. (2007) mentioned that MeHg contamination in fisheries from centuries of industrial atmospheric emissions negatively impacts humans and wildlife worldwide, and they also found that atmospheric settled MeHg does not stay in water for long periods, but preferentially enters into fish bodies during feeding [32]. 

The reason why Hg accumulates in fish as MeHg lies in its absorption and the metabolic mechanism of fish. Most forms of Hg eventually convert to MeHg or dimethylmercury (DMM) in fish due to methylation by the action of relevant microorganisms (anaerobic microorganisms) [33]. For instance, inorganic Hg can be transformed into MeHg and DMM by alkylcobalamin [34,35]. In short, elemental Hg present in water molecules is converted to MeHg by the action of microorganisms, and it eventually enters into fish bodies mainly through enrichment in the food chain [36].

## 3. Bio-Accumulation, Species-Specific and Geographical Differences in Mercury (Hg) in Fish

### 3.1. Bio-Accumulation of Hg in Fish through Food Chains

Hg, especially MeHg, is a typical compound that can experience biological amplification (i.e., bio-magnification) [37]. Regarding bio-accumulation, MeHg concentrations are magnified through the food chain, reaching concentrations in fish 10,000- to 100,000-fold greater than those in the surrounding water [38]. Being at the top trophic level, fish face serious risks of bio-accumulated contents of Hg through the food chain in water bodies [8]. Moreover, this bio-accumulation process is evident in fish species feeding at the higher trophic levels: Mediterranean tuna, anchovies, sardines and mackerel [39,40]. The observations at Lake Wisconsin in the USA found that the MeHg concentration (ng g^−1^) and the proportion (%) of MeHg to total Hg (THg) in the organic matter of phytoplankton, zooplankton and small fish were 34 and 18, 53 and 57, and 485 and 95, respectively; hence, the MeHg concentration and the proportion of MeHg to THg in organisms gradually increase with increasing trophic levels [41].

As top predators of the pelagic food web, some large fish and predatory fish naturally bio-accumulate Hg. The Hg concentrations in these fish are high, and the size of a fish is a determining factor of its Hg burden [42]. This is further supported by data from a survey of THg concentrations in Chinese marine fish, and the data from this study show that Hg levels in different fish species ranged from high to low: carnivorous fish (median: 58 ng g^−1^, range: 2.4–330 ng g^−1^), followed by omnivorous fish (median: 24 ng g^−1^, range: 6.0–155 ng g^−1^) and herbivorous fish (median: 16 ng g^−1^, range: 2.5–123 ng g^−1^) [43]. In addition to marine fish, the researchers also investigated freshwater fish, and the results showed the same trend [43,44]. Hg concentration is high in some long-living and predatory fish that simultaneously have higher opportunities for accumulating Hg [45,46,47].

### 3.2. Species-Specific Difference in Hg in Fish

Hg levels vary widely among different types of fish, and its concentration is affected by the specific physiological and ecological characteristics of different fish species [48]. In general, the Hg concentration in carnivorous fish is greater than that in omnivorous species [49], and there are high Hg concentrations in long-living predators (e.g., rockfish and sharks) [45]. Moreover, higher trophic-level fish (i.e., piscivores and carnivores) and benthic fish have higher mean THg concentrations [50,51]. Additionally, larger predatory fish contain the highest Hg concentrations, and Hg levels are positively correlated with body length, weight and age of the fish [52]. Vieira et al. (2021) indicated that carnivorous species presented higher Hg contents (range 0.03–0.88 μg g^−1^) when compared to omnivorous fish (range 0.003–0.19 μg g^−1^), demersal fish exhibited higher Hg levels (range 0.01–0.88 μg g^−1^) than pelagic species (range 0.003–0.38 μg g^−1^), and *Zeus faber* presented high Hg levels (0.68 ± 0.07 μg g^-1^) above the maximum limit (0.5 μg g^-1^) established for human consumption [53]. While several factors affect the Hg levels in fish, including nutritional level, size, and age of the fish, the Hg levels are generally higher in fish with high nutrient levels [41,54,55], and a significantly increasing trend in Hg concentration with fish size has been observed for all species (except for European anchovy), suggesting Hg bio-accumulation throughout the life cycle [56]. Dang and Wang (2012) indicated that biokinetic variation could explain the size-dependent Hg accumulation in fish, and both size-related g (growth rate constant) and k_e_ (efflux rate constant) were the key drivers [57].

Among the different fish tissues, muscle was a major reservoir for Hg and contained the highest ratio of MeHg/THg, liver was the second important organ for Hg accumulation in most fish species, and intestine was a critical organ for Hg bio-transformation, with the proportion of MeHg to THg differing greatly among different fish species [58]. Additionally, the Hg concentration in fish muscle correlated with the length and weight of the studied fish species of *Sander lucioperca* and *Esox lucius linnaeus* (carnivorous), *Cyprinus carpio* and *Carassius auratus gibelio* (omnivorous) [49]. It was indicated that the average Hg level (0.17 ppm wet weight) in fish muscle was within the range known to adversely affect sensitive birds and mammals, and only 4% of Pacific cod samples contained more than 0.5 ppm of Hg [52]. Additionally, the regression models in a study of cod (mostly from the Pacific Ocean) showed that 27% of the Hg variation was due to tissue examination and the age of fish, and the Hg levels in muscle were significantly higher than those in liver [52]. Moreover, although male fish not only ingest Hg at a higher rate than females, they also eliminate Hg at a higher rate than female fish [59]. Thus, sex, in contrast, did not influence Hg levels, suggesting that female and male fish have similar feeding habits [42].

### 3.3. Geographical Differences in Hg in Fish

Hg levels in fish vary widely from place to place, and a significantly positive relationship between the mean total Hg (THg) in fish and the latitude of the sampling site was observed in 40 different water bodies located in 26 countries [50]. High Hg levels occur near sources of Hg release from industrial (e.g., Minamata) and natural (e.g., Cinnabar) mines in the Mediterranean, and volcanic sources near Madeira. The Mediterranean basin contains large amounts of cinnabar sediment, which is why marine organisms here have a higher Hg burden than the same species living in other oceans [60]. Over time, it was experimentally determined that tuna caught in the Mediterranean had three to four times as much Hg as tuna from the Atlantic [60]. Moreover, significant differences were observed in the Hg levels of *Scomber australasicus*, *S. japonicus*, *Trachurus trachurus*, *Decapterus punctatus* and *Paralichthys olivaceus* between the Pacific Ocean and the Sea of Japan [61].

In China, Hg in fish is generally accumulated at a low level, but significant geographical differences were evident and formed hot spots from the north to the south [43]. For terrestrial aquatic ecosystems in China, the Hg content in fish is high in the north and low in the south, which may be related to the relatively high Hg emissions in Northern China [21]. However, the Hg content in most river fish did not exceed the national standard of China (0.3 mg/kg) or the international standard of WTO (0.2 mg/kg). Moreover, in terms of marine ecosystems, the fish in the Southern Sea contained higher Hg content than those in the North Sea, and the Hg content in sea fish (0.09–0.36 mg/kg) was generally higher than that in river fish (less than 0.1 mg/kg) [21]. In addition, data on Hg levels in fish from other regions of the world clearly demonstrated that the Hg content in sea fish was below 10.9 µg g^−1^ in North America [62], between 50 and 3100 ng g^−1^ in the Mediterranean, and between 10 and 1240 ng g^−1^ in the Western Indian Ocean [63]. This shows that there is some variability in Hg levels in fish from different regions.

In summary, the differences in Hg levels in fish are related to the differences in fish species and the different positions of fish in the biological food chain (mainly reflected in the level of Hg bio-accumulation in fish), but also depend on the differences in the nutritional status and age of individual fish, which are less dependent on the levels of Hg contamination in the environment [64,65].

## 4. Toxic Effects of Mercury (Hg)-Contaminated Fish on Pregnant Women and Fetuses

### 4.1. Background of the Toxic Effects of Hg Contamination in Fish on Fish and Humans

For fish, Hg markedly affects their physiological health even at lower exposure levels, and this is manifested microscopically in genetic mutations, tissues and physiology, and macroscopically in the survival, growth and developmental status of fish [48]. In addition, Hg exposure can produce teratogenic and neurotoxic effects, and reproductive toxicity, and these effects can then cause harm to cells, tissues, proteins and genes, and ultimately to the survival, growth and behavior of marine fish [48]. There are distinctly individual differences in the factors known to influence the hazard levels of Hg exposure [66]. Thus, there might be non-negligible differences in the effects of Hg toxicity on different individuals, species and life stages of fish [67]. Humans can be exposed to Hg, i.e., organic Hg (MeHg/ethyl-Hg), via the consumption of fish and poultry products, the use of insecticides, fungicides and pesticides, and in the forms of air pollution, medical equipment (e.g., thermometers and dental amalgam), certain vaccines, etc. [68]. The Hg that people usually ingest through eating fish is organic Hg [25,48]. The most important source of exposure to organic Hg in human beings seems to be the consumption of fish contaminated with MeHg [69]. MeHg bioaccumulates to differing degrees in various fish species and can have serious adverse effects on the development and functioning of the human central nervous system (CNS), especially during prenatal exposure [70]. Additionally, the harm of MeHg mainly lies in its toxicity to human nerves, and specifically the brain, which are most vulnerable to Hg [71]. The World Health Organization (WHO) estimates that the critical blood Hg concentration for MeHg poisoning is 200 μg·L^−1^ [72]. High-level exposure to Hg can result in significantly neurological and behavioral disorders, including tremors, memory loss, neuromuscular changes, renal and thyroid disorders, and even death [73]. Additionally, the body burden of Hg has been linked to hypertension in populations exposed to high Hg levels, and significantly positive associations between Hg and hypertension, and between Hg and blood pressure (BP) have been identified [74].

Hg’s toxic effects will differ depending on whether they have been caused by exposure to elemental, inorganic (as salts) or organic Hg compounds, and each form of Hg has a unique toxicological profile, and differs in the mechanisms of transport and disposition in human bodies. Exposure to inorganic and organic Hg can lead to adverse effects, including developmental toxicity, immunotoxicity, neurotoxicity, teratogenicity, and especially cytotoxicity, cardiovascular toxicity, hepatotoxicity and nephrotoxicity, disrupting endocrine systems and metabolic effects for human beings; all of these possible adverse outcomes from Hg exposure depend on the dose and length of Hg exposure, the Hg form, and the age and sex of the exposed human [17,18,19]. Both MeHg and vapor Hg are highly reactive and interact mainly with thiol-based proteins (-SH) in human bodies. MeHg exerts some toxic effects through altering protease activity, and both metallothioneins and glutathione appear to have a strong relation to the cytotoxicity caused by inorganic and organic Hg, respectively [75]. Moreover, MeHg affects several biological processes: it increases lipid peroxidation, generates reactive oxygen species (ROS), depletes glutathione (GSH), reduces cell membrane integrity, alters cell signaling and mitochondrial impacts, changes DNA repair and immunomodulatory impacts, affects the regulation of Ca^2+^, causes glutamate and calcium dyshomeostasis, and changes DNA methylation, which in turn have adverse effects on humans [17]. Balali-Mood et al. (2021) also indicated that Hg can disrupt cellular events including growth, proliferation, differentiation, damage-repairing processes and apoptosis, and the mechanisms of their action induce toxicity including ROS generation, the weakening of antioxidant defense, enzyme inactivation, oxidative stress, apoptosis, and caspase activation, as well as ultrastructural changes in hepatocytes, which have also been seen due to Hg exposure [18]. Renu et al. (2021) indicated that Hg can induce apoptosis in the liver; through the epigenetic mechanism, Hg can cause DNA methylation and disruption to post-transcriptional modifications [19]. Additionally, Bridges et al. (2017) indicated that the modulation of neurotransmitters, including dopamine and serotonin, in the brain may result in changes in behavior related to Hg exposure [76]. Furthermore, high exposure to Hg can deplete the amount of cellular selenium available for the biosynthesis of thioredoxin reductase and other selenoenzymes that prevent and reverse oxidative damage which, if the depletion is severe and long-lasting, results in brain cell dysfunction that can ultimately cause death [77]. Although multiple mechanisms of toxic action of Hg were discussed in [16,17,18,19,75], many aspects are still far from being satisfactorily understood.

### 4.2. Toxic Effects of Hg Contamination in Fish on Pregnant Women and Fetuses

It is undeniable that the intake of fish during pregnancy is beneficial for the body due to the diverse nutrients it contains. Numerous studies have shown that fish is rich in long-chain omega-3 polyunsaturated fatty acids (LCn-3PUFAs) and vitamins A, D and B_12_, which play an important role in the physiological metabolism [78,79]. However, aquatic organisms, mainly fish, are contaminated with numerous toxic substances, including Hg and other heavy metals and drug residues, which may have adverse effects such as teratogenicity [80,81]. Numerous statements have been made in the medical literature and by the WHO in the past, recommending that pregnant women limit seafood intake to avoid exposure to the potential toxicity of aquatic products [9]. Hg exposure in pregnancy has been associated with both pregnancy complications and developmental problems in infants [82]. In pregnant women, Hg passes through the placental membrane, which can cause spontaneous miscarriage, premature birth, congenital disability and irregular fetus development [83]. Additionally, even small amounts of fish consumed by mothers during pregnancy can cause elevated Hg levels and affect children’s neurobehavioral development, including basic skills such as listening, reading, and writing [84,85]. Meanwhile, in contrast, no negative effects of maternal fish consumption during pregnancy on local children’s neurobehavioral development were found in the Republic of Seychelles [86,87]. However, relevant indicators showed that the mothers on the Faroe Islands, who consumed mainly whale meat and blubber, had approximately 10 times more MeHg than Seychelles fish [87]. The difference in MeHg content undoubtedly contributes to the difference in findings, which also confirms in a comparative way the risk of MeHg for the fetus in pregnant women. Moreover, researchers measured blood Hg levels using atomic absorption based on 200 cases of deliveries in a Chinese hospital, and the incidence of fetal malformations, adverse pregnancy outcomes, hypertensive disorders of pregnancy, intrauterine growth irregularities, and fetal distress were found to be higher in the group with elevated blood Hg than in the group with normal blood Hg [88].

For pregnant women, the blood levels of Hg often exceed acceptable international levels, and the average Hg levels in the blood of mothers with premature births, low birthweight and spontaneous miscarriages were 30% higher in comparison with unexposed women. A significantly increased risk of premature birth, the birth of children with low body weight, and spontaneous miscarriages was also found when the Hg concentration exceeded 2 µg/L of plasma [89]. MeHg is usually absorbed by the body through the skin mucosa and the respiratory and digestive tracts; the most important route is the digestive tract [90]. As a common food item on the human table, the entry of MeHg into the body of pregnant women through the gastrointestinal route is often an important cause of Hg hazards [88]. After entering the bloodstream, Hg binds to the sulfhydryl group of hemoglobin and enters the organs of the body; subsequently, the amount of MeHg in the organs and tissue remains relatively constant [91]. It is noteworthy that the toxic effects of MeHg on the liver and kidneys are lower; the Hg levels in the organs and tissues are, in descending order: liver > brain > kidney > blood [90]. Additionally, the toxic effects of MeHg in the brain and the nervous system are relatively high [92]. The reason for this is, on the one hand, that brain tissue is rich in lipid-like substances that have a strong affinity for MeHg, and can easily enter brain tissue through blood flow; on the other hand, MeHg is strongly bound to the carbon–Hg chains in the molecular structure of MeHg, so MeHg can remain in brain cells for a long time and cannot be easily excreted [92]. The clearance of MeHg from the brain is delayed by 20% compared to other parts of the body, and MeHg accumulation in the brain is higher than that in the sensory and motor areas, especially in the posterior lobe of the brain [92]. The mechanism of Hg following injury to the mother involves several complex aspects of the body’s metabolism. Hg readily binds to sulfhydryl groups and enzymes in proteins, leading to the dysfunction of several enzymes in the body, including ATPase, lactate dehydrogenase, cytochrome oxidase and alkaline phosphatase, leading to severe enzyme inactivation [91]. Hg can also disrupt the structural integrity of genetic material by binding to multiple groups (e.g., hydroxyl and amino groups) in genetic materials (e.g., DNA and RNA), which can lead to DNA breakage and mutation in severe cases [88]. Additionally, Hg exposure often leads to visual field contraction, motor ataxia, dysarthria, tremors, cardiovascular diseases, etc. [93,94]. Hg and MeHg can cause mitochondrial dysfunction, decrease ATP synthesis, deplete glutathione, and increase phospholipids, protein and DNA peroxidation [95]. The vascular effects of Hg also include many aspects, such as increased oxidative stress and inflammation; decreased oxidative defenses; mitochondrial dysfunction; depolarization; autoxidation of the inner mitochondrial membranes; and the inactivation of oxygen phosphatase [71].

For the fetus, once incorporated into the body, MeHg easily penetrates the blood–brain barrier (BBB) and causes damage to the central nervous system (CNS) [20]; high blood Hg levels can increase the incidence of intrauterine growth irregularities and fetal distress [96]. In general, Hg can pass through the placenta into unborn infants, and early exposure to Hg is correlated with infant health effects, such as neurological, developmental and endocrine disorders [97]. The teratogenicity of MeHg and its effects on fetal growth and development have also been confirmed in a trial of singleton pregnancies [88]. Gilbertson (2004) indicated that perinatal exposure to MeHg is known to result in severe neurological effects on the developing fetus and infant, including cerebral palsy, intellectual disability, and seizures [98]. Simultaneously, Hg exposure is also extremely harmful to the fetus, causing cardiovascular disease, hypertension and changes in heart rate variability [99,100]. Since cardiac rhythm and function are controlled by the autonomic nervous system, and it has been hypothesized that the neurotoxic effects of Hg may also affect cardiac autonomic function [101]. Exposure to Hg may have long-term effects on the cardiac parasympathetic activity of children. The intrinsic mechanism that makes elemental Hg is so damaging to the nervous and cardiovascular systems is due to the high affinity of Hg for sulfhydryl and selenium groups, which are present in glutathione precursors such as cysteine [101].

### 4.3. Interactive Toxic Effects of Hg Contamination in Fish on Pregnant Women and Fetuses

The main reason for the widespread concern about fish intake by pregnant women is the vulnerability of the pregnant woman and the fetus itself, and the hazard extent of Hg exposure [102]. The fetus is relatively vulnerable to adverse external factors, and the incomplete development of the fetal liver results in the inability to excrete toxic substances and pollutants in a timely and effective manner [103]. The dangers of Hg are also passed between the pregnant woman and the fetus, and affect each other [102]. On the one hand, pregnant women who consume a large amount of fish might have elevated blood Hg levels, and the Hg level in cord blood is much higher than that in maternal blood [9]. Kim and Kim (2006) also indicated that the blood Hg content in the umbilical blood is substantially higher, and this may lead to higher blood Hg levels in neonates [9]. Higher Hg concentration in the fetus compared to that in the mother may affect immature fetal organs [104]. Additionally, the placenta in pregnant women does not present a barrier to Hg and the fetus has a high accumulation capacity for MeHg, with 1.8–4.0 times the MeHg content in the brain, liver, kidney, heart and lung than that of normal adults [105]. On the other hand, MeHg has lipolytic properties with a strong affinity for lipid-like substances and can easily pass through membranous tissues, such as the placental barrier and the blood–brain barrier (BBB), and it is easy to cause direct all-round damage to the fetus [20]. Because of its lipid solubility and short-chain hydrocarbon structure, Hg can rapidly pass through the placenta and be oxidized into ionic complexes that bind with high affinity to fetal hemoglobin, and cannot be returned to the pregnant women’s blood circulation [38,83]. Moreover, Hg easily binds to sulfhydryl groups, so proteins and enzymes containing sulfhydryl groups (e.g., ATPase and lactate dehydrogenase) are disturbed or even inactivated by the binding of Hg [91]. Additionally, genetic material containing amino and phosphate groups (e.g., DNA) can be damaged by Hg binding [88].

**Figure 2:**
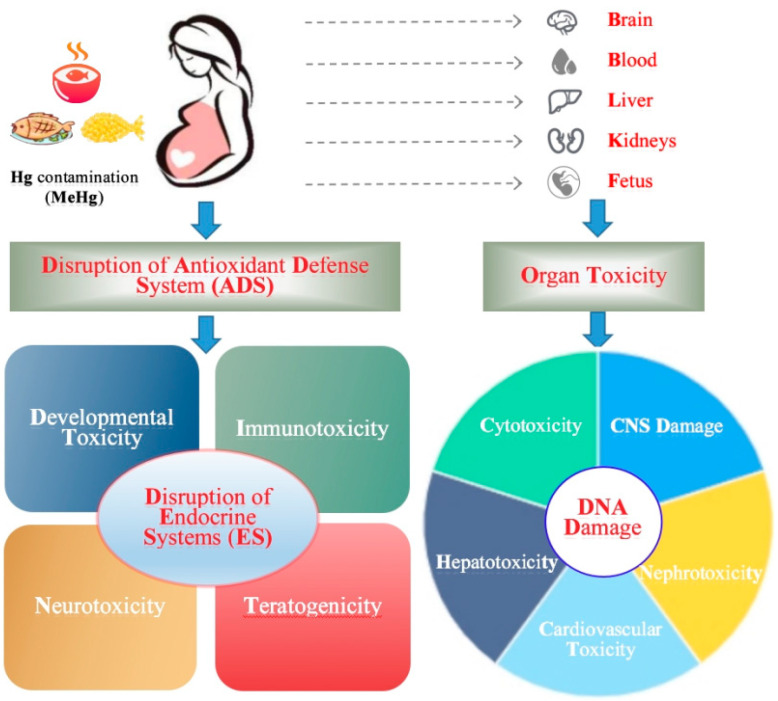
Action model of high exposure to mercury (Hg) for pregnant women and fetuses through consumption of Hg-contaminated fish. Note: ADS—antioxidant defense system (including various enzymatic and nonenzymatic antioxidants); ES—endocrine systems (i.e., glands) which produce and release different hormones. CNS—central nervous system; For the CNS, Hg can inhibit the formation of myelin to prevent nerve sheaths from forming properly. Blood and brain—the blood–brain barrier (BBB) against toxic chemicals. MeHg easily penetrates BBB and causes CNS damage, particularly in fetuses. Liver—Hg can induce apoptosis in the liver through DNA damage by disrupting DNA methylation and disrupting post-transcriptional modifications. Adapted from Balali-Mood et al. (2021) [18].

Based on the toxic effects of Hg-contaminated fish on pregnant women and fetuses, and in combination with the reviewed multiple mechanisms of Hg’s toxic action on humans [16,17,18,19,75], an action model of high exposure to Hg for pregnant women and fetuses through the consumption of Hg-contaminated fish is elucidated in Figure 2, adapted from Balali-Mood et al. (2021) [18]. It is elucidated that high exposure to Hg for pregnant women and fetuses is harmful, and has many adverse effects on various organs (especially the liver, kidney and brain), and disruption of the antioxidant system may play an important role in Hg’s toxic effects. Simultaneously, signaling transduction, protein or/and enzyme activity and gene regulation are involved in mediating toxic and adaptive responses to Hg exposure. Information on the mechanism involved in Hg toxicity is growing, but knowledge gaps still exist between the adverse effects and mechanisms of action, especially at the molecular level.

## 5. Recommended Fish Diet for Pregnant Women Based on Toxic Effects of Hg Contamination in Fish 

Fish is considered a healthy food with exceptional properties, as it is rich in vitamins, minerals, high-quality proteins and essential 3FAs [1,2,3,4,5]. During the pregnancy period, pregnant women need more nutritional supplements than ever before. However, given the toxic effects of Hg contained in fish products, there is often a “trade-off” for them in fish consumption to achieve a relatively favorable ratio [6,104,106,107]. The rational consumption of fish is a must in terms of nutrient intake to ensure the health and safety of pregnant women [11,108]. On the one hand, more innovative and new fish products need to be developed to better suit the seafood intake needs of pregnant women. On the other hand, a proper understanding of fish consumption needs to be further promoted and popularized in general, especially for pregnant women. Some researchers recommend regular fish oil supplementation during pregnancy [109]. Although many reports have elucidated the benefits of fish oil [110,111], some studies have concluded that it is not beneficial, or have even reached the opposite conclusion [112,113,114]. Given the effects of high doses of cod liver oil on hypertension in pregnancy [113], and some adverse effects of MeHg arising from the consumption of regular fish oil [115], the rational recommendation of dietary fish intake is a must due to the complex interplay between MeHg and fish-oil-derived fatty acids [116]. Fish oils come from different fish species and involve some variation in contamination status and origin purification level; therefore, the most conservative recommendation is to consume a variety of low-Hg-contaminated fish for health benefits [117].

Additionally, some researchers recommend eating small fish because they have low bio-concentration levels of toxic substances such as Hg in their bodies [44,57]. Additionally, avoiding specific species (mainly carnivorous fish), limiting the intake of fish for pregnant women, avoiding the intake of fish from heavily polluted waters, and the selective intake of aquatic products (e.g., shellfish and shrimp) is also recommended. The importance of fish as a food should not be overlooked because of the toxic effects of harmful substances (including Hg), and the benefits of its important nutrients need to be properly and widely disseminated to the consumer community, especially pregnant women. In a study on the effects of fish consumption and fetal neurodevelopment in women of childbearing age, the authors stated that fish consumption in dietary intake should focus on nutrients, such as docosahexenoic acid (DHA), as mentioned by Mendivil (2021) [4]. The population of pregnant women is encouraged to consume fish with high DHA and low MeHg, such as anchovy, Arctic char, Atlantic mackerel, catfish, cod, haddock, herring, perch, pollock, salmon, sardines, shellfish, tilapia, trout and tuna, etc., and strictly avoid consuming fish with high MeHg levels (such as bluefish, croaker, eel, king mackerel, shark, swordfish, tilefish and weakfish, etc.) (as described in Table 1).

As for the frequency and amount of fish consumption, the relevant safety and health authorities have given different recommendations based on different countries and regions. For instance, in March 2004, the Department of Health and Human Services (HHS) and the Environment Protection Agency (EPA) of the USA published a report entitled “What You Need to Know about Mercury in Fish and Shellfish”, which showed that nearly all fish and shellfish contain trace amounts of Hg. Thus, to avoid Hg contamination, in 2010, the U.S. Department of Agriculture (USDA) and the U.S. Department of HHS recommended the public to consume no more than 8 ounces (227 g) of a variety of seafood per week, which equates to an average daily intake of 250 mg of fatty acids, including DHA [120]. In addition, the US EPA recommended that total blood Hg concentration should remain lower than 5.8 µg/L for women of childbearing age [121]. The British Food Standard Agency (FSA) recommended that pregnant women, women of childbearing age, and children under 16 years of age should avoid consuming swordfish and tuna (which are higher ranking in the ocean food chain) because of their high Hg content, and recommended that pregnant women and women of childbearing age should avoid consuming more than two tuna steaks per week [72,122,123].

Many documents have also presented fish consumption views and guidelines for pregnant women [6,52,106]. However, these recommendations tend to be generalized in nature. Specifically, for individuals, the amount of fish recommended per week depends on the frequency and portion sizes of fish a person eats, and the individual’s physical condition, such as the individual’s sensitivity to toxicity, body weight, etc. More precise recommendations can be obtained from national or local public health departments [118]. In addition, based upon the study on the awareness of fish Hg, and in compliance with the WHO’s enforcement of fish consumption among pregnant women, researchers found that women with higher incomes and education and those living in coastal states were more likely to be aware of Hg in fish food, suggesting that information about safe fish consumption is not being communicated equally to all groups [124,125].

However, there have been many studies on the toxicity of MeHg in fish which have drawn opposite conclusions. Some claim that the risks of Hg effects caused by moderate fish intake are less than the beneficial effects of fish nutrients on the human body, and maintain a positive attitude towards fish intake as a whole [126]. Gale et al. (2008) indicated that, as oily fish is a major dietary source of 3FAs, it is possible that a low intake of fish during pregnancy may have adverse effects on the developing fetal brain [127]. Many experiments have shown that Hg does not affect pregnant women and fetuses in small amounts [10,108,128,129]. No significant conversion of Hg species was observed after fish cooking treatment; an overall loss of up to 33% of Hg species in fish was observed after frying, and most of the Hg lost during the cooking procedure came from CH_3_Hg^+^, so it was concluded that the fish diet was neutral, especially after cooking treatment [130]. There is no consensus on the effects of fish containing MeHg on pregnant women or the neurological effects on the fetus. Persistent chemical pollutants may bio-accumulate and have the potential to achieve teratogenic or other adverse effects. In addition, the ultimate consequences of exposure to toxic chemicals (including Hg) in pregnant women, especially in the long term, are uncertain [131]. Therefore, some studies concluding that exposure to low levels of chemical toxins (including Hg) during pregnancy has no long-term effects are incomplete and inappropriate, and more high-quality precise and scientific studies are warranted in the future.

A low-Hg dietary intake of fish per week (where women consume less than 6 ounces, equivalent to the WHO’s recommendation of 170.1 g per week) is the current existing recommendation. However, the average fish intake for the female population ranges from 89 g to 120 g (2 to 3 ounces) per week or less. The overall fish consumption pattern of females is as follows: pregnant women > postpartum women > normal groups of women [128]. This finding is similar to the FDA’s analysis of fish consumption, which estimated the average fish consumption of all women aged 16–45 to be 13.4 g per day (i.e., 93.8 g per week). The 2003–2004 NHANES results estimated that women aged 16–45 years averaged 10.3 g fish per day [132]. These data respond to a phenomenon that indicates a deficit in the promotion of fish consumption. Predominantly pregnant and postpartum women appear to be following national or local safety and health organization recommendations of “no eating”, rather than maintaining a regular intake of low-Hg fish. This results in pregnant women not consuming enough low-Hg fish to benefit their health [125,128]. Therefore, education and media coverage for pregnant women needs to be further improved, and the government and society need to take measures to avoid extreme attitudes toward fish consumption among pregnant women, i.e., total ban or no concern at all. Agencies need to adequately communicate the benefits of consuming adequate amounts of low-Hg fish food, while in the meantime raising awareness among pregnant women about the dangers of Hg contamination in fish and other fish products, as per described in this review. Moreover, metallothioneins and glutathione appear to have a strong relation with inorganic and organic Hg cytotoxicity, respectively [75]. Hair is considered as an index of Hg exposure, since MeHg accumulates there (the average ratio of hair-to-blood concentrations of MeHg is about 250:1) and Hg is excreted in urine and feces [76]. Thus, hair, urine and feces, and some specific proteins of pregnant women, can be used as special biomarkers for early warnings of Hg contamination in the daily intake of fish in the diet.

## 6. Conclusions and Perspectives

As one of the top ten chemicals or groups of chemicals of major public health concern, Hg is continuously discharged from natural sources and industrial activities. The health effects of Hg contamination on humans, especially pregnant women (including fetuses), who are susceptible to Hg (especially MeHg) exposure, even though at low levels, has become a worldwide concern. This review paper described Hg forms in nature and fish bodies, as well as the bio-accumulation of Hg in fish through the food chain in the water ecosystem, and further reviewed the interactive toxic effects and action mechanisms of Hg-contaminated fish on pregnant women and fetuses. Based on the knowledge that inorganic Hg cannot pass the blood–brain barrier (BBB) or placenta, and liquid Hg^0^ is only slightly absorbed in the gastrointestinal (GI) tract, these two forms of Hg do not appear to be toxic, while vapor Hg^0^ and organic Hg (including MeHg) have high toxicity even at low levels because they can pass the BBB (blood–brain barrier) and cause CNS (central nervous system) disorders. Because there are species-specific and geographical differences in Hg bio-accumulation in fish, this review paper provides practical recommendations for people, especially pregnant women, to select the right species and specific tissues of fish and seafood with low concentrations of Hg, and suggests cooking fish. In this review paper, the important public health dilemma of whether pregnant women should eat or not eat fish exposed to mixtures of healthful nutrients and Hg contamination was addressed. In the future, based on the accurate measurement of the Hg content in different species of fish in the corresponding waters of different regions and the detailed classification of different populations of Hg-contaminated fish in the region, local governments and health organizations should further provide, more accurate and personalized fish dietary intake recommendations for specific populations, such as childbearing-age women who might become pregnant or are pregnant, nursing mothers, and young children under 16 years of age, so as to ensure the maximum benefits of fish dietary intake.

## Figures and Tables

**Table 1:** Choice guidelines for species and types of fish based on Hg contamination for daily fish consumption for pregnant women.

Choices	Level of Hg Contamination	Types of Fish	Species of Fish	Cited References
Right	Low Hg/MeHg	Freshwater fish, herbivorous fish, small fish, etc.	Anchovy, Arctic char, Atlantic mackerel, catfish, cod, haddock, herring, perch, pollock, salmon, sardines, shellfish, tilapia, trout, tuna, etc.	[42,43,44,45,46,47,49,50,51,52,53,107,118,119]
Wrong	High Hg/MeHg	Marine fish, piscivores and carnivores, benthic fishes, large fish, predatory fish, etc.	Bluefish, croaker, eel, king mackerel, shark, swordfish, tilefish, weakfish, etc.	
